# Assessment of Animal Welfare at an Exotic Animal Fair in Poland: A Focus on the Quality of Exhibition Containers for Reptiles and Amphibians

**DOI:** 10.3390/ani14131872

**Published:** 2024-06-25

**Authors:** Damian Zieliński, Piotr Nawłatyna, Zofia Wójcik, Barbara Sobieszek, Arkadiusz Słaby, Martyna Nolewajka, Joanna Kapustka

**Affiliations:** 1 Department of Animal Ethology and Wildlife Management, University of Life Sciences in Lublin, Akademicka St. 13, 20-950 Lublin, Poland; piotr.nawlatyna@gmail.com (P.N.); zwojcik_4a@wp.pl (Z.W.); arkadiusz.slaby1992@gmail.com (A.S.); joanna.kapustka@up.lublin.pl (J.K.); 2 Student Research Club of Animal Behavior and Welfare: Terraristics Section, University of Life Sciences in Lublin, Akademicka St. 13, 20-950 Lublin, Poland

**Keywords:** animal welfare, exotic pet trade, reptile, amphibians, responsible herpetoculture

## Abstract

**Simple Summary:**

The growing interest in exotic animals makes exotic animal fairs a popular place for trading. However, there are concerns about the welfare of these animals. This study seeks to evaluate the well-being of the reptiles and amphibians available at exotic fairs in Poland (based on photos of the display boxes). The goal is to identify any existing issues and propose potential improvements to enhance these animals’ presentation conditions. This study analyzed photos of temporary containers for exotic animals presented at a fair, focusing on the size of the containers, presence of substrate, availability of environmental enrichment, the occurrence of visual abnormal postures and behaviors, and an overall welfare assessment.

**Abstract:**

Given the growing number of events involving exotic animals, it is crucial to prioritize the well-being of the animals involved. This study aims to evaluate the quality of animal presentation at a selected fair in Poland and assess the level of animal welfare evident in the exhibition boxes, contributing to the ongoing dialogue on this important issue. The evaluators used a five-point Likert scale and a Yes/No system to analyze the living conditions during the fair, including the size of containers, presence of substrate, and environmental enrichment. They also assessed the occurrence of visual abnormal postures and behaviors to gauge the overall level of welfare. To ensure the reliability and consistency of the data and minimize potential bias, each evaluator repeated the rating process three times, with a three-week interval between each session. An average value was then calculated for each aspect. A total of 818 animals were present at the fair, with 688 being reptiles (84.11%) and 130 being amphibians (15.89%). This study revealed that the provision of substrate scored higher for reptiles compared to amphibians, while the size of containers for amphibians received higher ratings than those for reptiles. Visual abnormalities in posture and behavior were more common in reptiles than in amphibians. Display containers for snakes received the lowest ratings and showed more visual abnormalities in posture and behavior, raising concerns about their welfare. Despite the presence of environmental enrichment, the overall level of animal welfare was assessed as being medium/low. Pearson’s correlation coefficient indicated good reliability among the evaluators during the assessment process, with most assessments showing values > 0.8. Despite existing regulations for exhibitors, neglect remains prevalent. These findings highlight the potential negative impact of animal exposure at fairs on animal welfare. Display containers were often inadequately sized for the animals, particularly for snakes, chameleons, monitor lizards, and salamanders.

## 1. Introduction

The responsible husbandry of exotic animals in captivity involves creating environmental conditions that are adapted to the species’ biology. This includes aspects such as temperature, humidity, UVB lighting, nutrition, and the size of the enclosure [1,2,3,4]. Responsible keepers and breeders arrange living spaces in a way that allows animals to manifest a natural range of behavior (through the use of environmental enrichment and novel objects, e.g., [5,6,7,8] and hide from the view of humans [4,9,10]. Adhering to the concept of quality of life [11], animals should have the best possible conditions in captivity if kept as pets. However, these aspects are often overlooked and not properly taken into account when purchasing animals at exotic animal fairs. It is important to provide the best possible conditions for animals kept as pets. 

### 1.1. Animal Welfare during Exotic-Pet Fairs 

**Figure 1:**
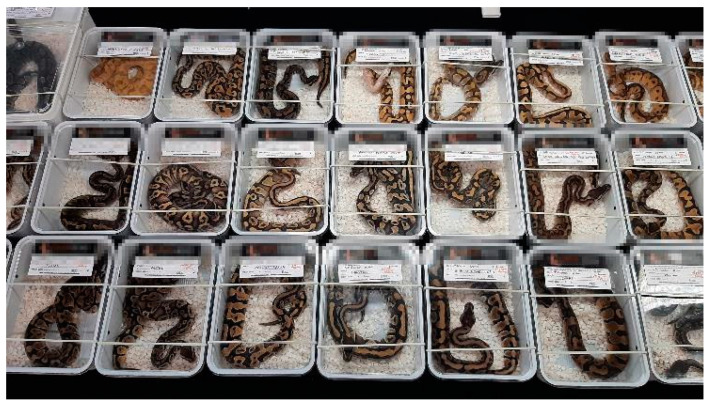
Display of Ball Python Snakes (*Python regius*) at the Fair (Breeder’s identity concealed).

Exotic animal fairs involve direct contact between buyers and sellers, potential owners, and animal breeders. Vendors display animals in small plastic containers grouped next to each other on tables to present the specimens they breed and raise or import [12,13]. However, considering the welfare of the reptiles and amphibians of interest in this study, one must wonder whether such a method is appropriate. In theory, they only spend a few hours being displayed at the fair (which can be considered short-term accommodations). However, transportation and preparation time should also be considered [14]. Vendors participating in such events arrive from all over the country and sometimes from abroad, which sometimes results in the animals being stored in a container for several days. Furthermore, the animals may have to travel further, returning unsold or with new owners. In addition, some events operate over two days, and animals remain confined. Therefore, considering the time required for preparation, transportation, the duration of the fair, and the return trip, it is unreasonabke ti view this as a short-term event. Furthermore, this period is associated with severe stress, with the animals being kept in cramped spaces, transported, displayed on a table, in contact with a unlimited number of visitors, and confined in unsuitable environmental conditions (usually, the temperature is not suitable for most common species). Temperature plays a key role, as reptiles and amphibians are cold-blooded animals [12,15]. If the air temperature is too low or too high, they can not thermoregulate properly, leading to discomfort and illness [1,16,17]. At fairs, animals are housed primarily in small plastic containers that sometimes do not even allow them to stretch (Figure 1). Animals housed in larger enclosures are less stressed and more active [18,19,20]. Most reptiles and amphibians are prey animals, so they should be provided with somewhere to hide (or so-called environmental enrichment that allows hiding during the fair).

Natural behavior consists of exploring, locomotion, foraging, social behaviors (affiliative or agonistic), reproductive and parental, and (in some cases) territorial behavior [21]. At expos, reptiles and amphibians are housed in small plastic boxes, usually bedded with paper towels or loose, unnatural substrates, such as wood chips or lignocel, with no extra enrichment. It is believed that poor husbandry is the leading cause of most diseases in reptiles [22], and the same is true for amphibians since their care requirements, housing, and diet are similar. Stressors inhibit the functioning of the immune system and cause many physiological changes in the body [23]. At exotic pet expos, stress and fear indicators are expected, such as hyper or hypoactivity, hyperalertness, rapid movement or freezing, hyperventilation, open mouth breathing, self-aggression or aggression towards other animals or humans, etc. [10]. 

### 1.2. Public Health and Safety

Exotic animal fairs attract many enthusiasts and lovers of exotic species. However, during these events, many potential threats must be considered [24,25]. During exotic animal fairs, many people can directly contact various animal species. Unfortunately, not everyone adheres to proper precautions, such as biosecurity measures. As a result, there is a risk of transmitting zoonoses—infectious diseases transmitted between animals and humans [26]. Examples of such diseases include salmonellosis [27,28], chlamydiosis, babesiosis [29], and hantaviruses [30]. Fair participants must be aware of the risk of infections and follow appropriate hygiene practices [31]. Fair visitors can directly contact the animals, including handling and petting them. Unfortunately, some species can be aggressive or unpredictable, increasing the risk of potential bites or stings [25,32]. Individuals who have not handled a particular species should be especially aware of this risk and take appropriate precautions, such as wearing protective gloves [33]. Some participants at exotic animal fairs may have phobias related to specific animal species, such as spiders or snakes [34,35]. The presence of these animals at fairs can trigger strong fear reactions in some individuals, which can affect their comfort and safety. Therefore, fair organizers need to provide appropriate signage and separate areas where animals are present, allowing participants to avoid contact with those that cause them intense fear [36].

### 1.3. Potential Risk of Animal Escapes

Exotic animal fairs are often where rare and exotic species, which may not naturally occur in a particular region, are traded. There is a risk that some of these animals may escape or be inadvertently released due to improper practices, e.g., opening containers, taking out animals for photos, and exposing animals outside containers [31]. This can lead to alien or invasive species being introduced into the local ecosystem, which can negatively impact the local flora and fauna [31,32,33,37]. In addition, people with animal phobias, e.g., arachnophobia or ofidophobia, may tolerate animals when confined in boxes. Nevertheless, when animals escape or are let loose, they may panic, which can eventually spread to other participants of the fair [34,35]. Therefore, fair organizers should abide by proper safety procedures to minimize the risk of animal escapes.

### 1.4. Activities and Regulations of Exotic Pet Trading

In many European countries, exotic animals are exhibited for sale in dozens of events [37]. It is essential to look at the rules and requirements that organizers set for vendors to prepare conditions for animals during events. Offering animals at exotic pet fairs and selling them online is one of the most popular ways of trading such animals. Previous publications regarding vertebrates at exotic fairs indicate low levels of animal welfare [38,39], although the situation has begun to change in recent years. Organizers of such fairs started to require some basics that affected the conditions of exhibiting traded animals and offering pets. Indeed, terms and conditions depend on fair organizers and can differ.

All vertebrates offered must be displayed in opaque containers with transparent lids. Presumably, this prevents stressing out animals exhibited next to each other and creates a small, safe space for animals [36,40,41,42,43,44]. Aquatic species must be exhibited in aquariums, and wetlands species must be provided with suitably high humidity [41,42]. All vertebrates must be housed separately during fairs. The size of the container and given spaces should provide animals with the ability to stretch. In places where fairs occur, the temperature should be suitable for most exotic pets, but as observed, it depends heavily on the time of year. It might be too cold during autumn, winter, and spring, even considering the short exposure time. If necessary, the vendor must provide a suitable temperature by heating the tables [37,42].

Many of the animals offered at such fairs are protected species where they occur in the wild. They are also under international protection by being listed on annexes of the Washington Convention (CITES). Therefore, breeders are required to have documents proving the legality of the animals they are selling (legal import documents, or proof that the animals were born in captivity) [36,41,42,45]. Exhibiting and offering animals that are listed as dangerous to human health and life, as well as transgenic and those classified as alien or invasive in host and the European Union, is generally prohibited [36,42,45]. Trading certain snake and lizard morphs is also banned at some fairs due to specific health issues. For example “Spider” ball pythons, often suffer from wobble syndrome caused by neurological problems and the “Enigma” morph in leopard geckos also presents concerns [41,46,47]. According to the fair regulations, Visitors are also forbidden to bring live animals to fairs, take animals out of containers, and have them on their hands [31,36,43]. These measures protect animals from stress, infectious diseases, and damage caused by inexperienced visitors. The terms and conditions of fairs also include the registration of sellers to prevent unauthorized people from accessing animals.

The fundamental aspect is that all animals offered for sale must be healthy and in good physical condition. Organizers require minimum weight and age of specimens belonging to certain, popular species (e.g., *Eublepharis macularius*, 15 g; *Corelophus ciliatus*, 10 g; *Pogona vitticeps*, 40 g; and *Chameleo calyptratus*, 20 g [48]). All exhibited animals have to eat on their own [42,43]. All fairs in Poland where animals are exhibited must be inspected by a veterinarian [49]. Animals must be labeled with species names, which helps new owners determine and provide appropriate conditions for their new pets. Some fair rules require sellers to hand over details about keeping a traded animal, including, among others, the origin of the animal, the status of protection, information about food requirements, enclosure size, habitat, humidity, temperature, water, and UV light demand requirements [36,40,41,42].

However, despite the time that exhibited animals spend at fairs, transport also affects their welfare. As has been observed, some vendors travel from fair to fair and do not repack animals into larger enclosures in the often long intervals between fairs, even if they are held in different European locations. This leads to animals spending most of their time in “temporary” containers until they are sold. The rules of some fairs, like Terraristika Hamm and Terraria Houten, include transport conditions, but it is hard to control and verify them [41,42].

In our study, we aimed to see if the animals (reptiles and amphibians) at one fair in Poland were provided with the right level of welfare by properly selecting environmental conditions for the fair’s duration. In addition, our objective was to determine if a trained team could consistently assess the exhibition containers for specific criteria based on photographs, aiming to establish consistency between on-site raters at the fair and those evaluating images post-event.

## 2. Materials and Methods

This research study was based on photos of temporary containers taken during an exotic animal trade show in Poland. We decided not to name the organizer company, as currently, in Poland, there is a significant discussion about the regulations concerning exotic fairs, animal welfare, and the tremendous competition between organizers.

The authors were present at the exhibition hall before it opened to the public. During the hour before opening, when the exhibitors and vendors had their stands ready for the start of the fair, we took pictures of the animals in the boxes for later evaluation. A total of 818 animals were photographed, with their containers displayed on tables before the fair began. The photos were then evaluated in terms of each specimen’s welfare. Behavioral and welfare assessments based on animal photographs have also been performed in other studies on laboratory or farm animals [50,51,52]. The display containers in which the animals were offered for sale were photographed. However, this study only included the containers that were on the table, and it was not possible to include the other containers that were hidden under the sellers’ tables. Each picture was taken to provide detailed visual documentation of the enclosure, serving as the basis for subsequent evaluation.

Ten study expert raters underwent a preparatory phase where evaluators were assigned the task of reading and comprehending scientific publications related to the well-being of reptiles and amphibians, methods of keeping them in captivity, ways of assessing their welfare levels, and behavioral aspects linked to stress. A list of publications is included in Supplement S1. Afterward, training was provided for the testers on five selected parameters (size of the container, substrate, presence of abnormal behavior, availability of environmental enrichments, and animal welfare). The exact guidelines are listed in Supplement S1. After the training, an evaluation of knowledge and skills in correctly assessing the listed parameters was performed. Because of this parameterization, seven out of the ten expert raters qualified for the experiment.

A group of seven expert raters performed the evaluation. Firstly, the terms of the evaluations were discussed to ensure a consistent method for the testers. Evaluators had adequate knowledge of the biology, behavior, and breeding conditions of reptiles and amphibians in herpetoculture, as well as stress indicators in reptiles and amphibians and their welfare levels under captive conditions (see above, and Supplement S1). Each person evaluated the photographs individually to avoid the effect of suggestions from other evaluators. The evaluation included four features: the containers and their equipment, the behavior/position of the animals captured in the photo, and the fifth feature, the final evaluation. Each aspect, except for enrichments, was rated on a Likert scale with a range of 1–5, with 1 representing the lowest rating (indicating poor welfare) and 5 denoting the highest (indicating optimum welfare). In the case of visual abnormal behaviors and postures, the lowest score was for the absence of such images/behaviors in the photo, and the highest score was awarded when the abnormality could be easily recognized. Environmental enrichments were assessed on a Yes/No system (present or not).

The parameters evaluated were related to the following:

- The size of the container (how well was it adapted to the species and size of the animal, were the dimensions properly assigned, as well as container orientation: horizontal for terrestrial species and vertical for arboreal) [18];
- Substrate (type and thickness, adaptation of substrate type to the needs of the species, higher humidity for amphibians and tropical species, low humidity for dryland, steppe, and desert species) [17];
- The presence of abnormal postures and behaviors (any signs of welfare issues, e.g., being at the air vent, unnatural body position, and in the case of snakes, adjusting the body to the contour of the container) [10,53,54];
- The availability of environmental enrichment (for example, we evaluated the presence of places to hide and the possibility of climbing for arboreal species; the presence of substrate or paper towel was not considered enrichment) [22];
- An overall objective assessment of animal welfare (the evaluator’s impression of the animal’s overall welfare in that container offered for sale).

All group members independently rated each photograph, ensuring a comprehensive evaluation. To enhance the reliability and consistency of the data and minimize potential bias, each person repeated the rating process threefold, each time after a three-week interval. An average value was calculated for each aspect.

### Statistical Analysis

The statistical analysis of the results was carried out using the Statistica 13.3 statistical package [55]. The basic classical statistical measures of the arithmetic mean (M) and standard deviation (SD) were used to describe the distributions of the studied characteristics. The conformity of the distributions of the studied measures to the normal distribution was assessed using the Shapiro–Wilk test [56]. The results were considered statistically significant at a level of significance of *p* < 0.05. To compare the results of the assessment regarding particular tester and trial number, two-way ANOVA was used. To assess the significance of differences between pairs of group means, the post hoc Tukey’s Honest Significant Difference (HSD) test was performed. Test–retest reliability using the Pearson correlation coefficient (r) was used to measure the reliability of the test and refers to the extent to which a test produces similar results over time.

## 3. Results

### 3.1. Evaluation of the Quality of Exhibition Containers and Animal Welfare

A total of 818 animals were on the tables, of which 688 were reptiles (84.11%) and 130 were amphibians (15.89%). In total, 50.59% of the animals available for purchase were species listed in Appendix II of the Washington Convention (CITES). The information about the legal status of these animals should be placed on the box, which was not fulfilled by any of the sellers. However, the rules of the event require exhibitors to have documents proving the legality of the animals.

**Figure 2:**
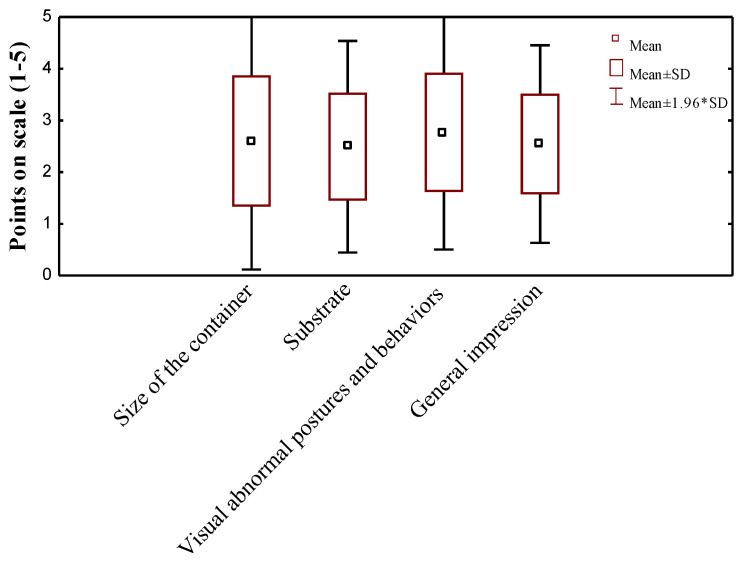
Evaluation scores (mean values) of animal display containers. Five-point Likert scale with one representing the lowest rating and five denoting the highest; for descriptive statistics, mean arithmetic (M) with standard deviation (SD) was used.

The results of the evaluations carried out by the testers of the exhibition containers in terms of characteristics related to animal presentation (size of the container and substrate), the presence of visible signs of abnormal behavior, and the general impression of the level of animal welfare in the container are summarized. Testers, given a five-point scale, rated the size of the containers used in which the animals were offered at 2.60 points (±0.009), the type and amount of substrate used at 2.49 (±0.007), and undesirable behavior at 2.77 (±0.008) (Figure 2). Their overall impression of how the animals were presented and their feelings about their welfare averaged 2.54 points (±0.007).

Analysis of the obtained scores considering the systematic division of animals shows statistically significant differences between reptiles and amphibians. The size of the amphibian containers was rated higher than those used for reptiles (F(1, 17,176) = 498.32, *p* < 0.001; mean 3.09 ± 0.02; 2.51 ± 0.01, respectively); meanwhile, the substrate (F(1, 17,176) = 341.72, *p* < 0.001) in the case of reptiles was rated higher (2.55 ± 0.01) than in amphibians (2.16 ± 0.02). Visually abnormal postures and behaviors (F(1, 17,176) = 278.04, *p* < 0.001) were more frequently observed in reptiles (2.71 ± 0.01) than in amphibians (3.11 ± 0.02), while the general welfare assessments in both groups were similar (F(1, 17,176) = 54.08, *p* < 0.001; 2.52 ± 0.01 in reptiles; 2.67 ± 0.02).

Dividing the animals into more precise systematic groups also shows apparent differences in the scores obtained (Table 1). Containers in which snakes were offered for sale were rated the lowest in terms of size, and the occurrence of visually abnormal postures and behavior and an objective assessment of animal welfare were rated the lowest (2.30 points on a 5-degree scale). Chameleons and salamanders were kept on the lowest-rated substrate (2.04 and 1.90, respectively). The perception of the level of welfare and the conditions in which the animals were displayed for sale was rated below 3 points in all groups of animals (ranging from 2.30 to 2.81 points). This indicates a relatively low level of welfare for these animals.

**Figure 3:**
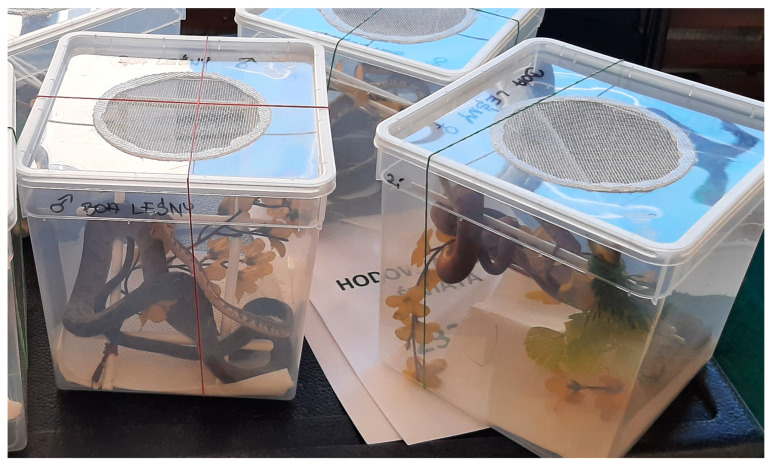
Display containers for arboreal snake *Corallus hortulanus* with environmental enrichment, providing the ability to climb.

Only 3.95% of the animals on display had additional environmental enrichment (not including the substrate) in the container, in the form of, for example, branches for arboreal species (Figure 3), artificial leaves, etc. These included chameleons (74.19% of all chameleons), snakes (2.90% of all snakes), and lizards (0.31% of all lizards).

**Figure 4:**
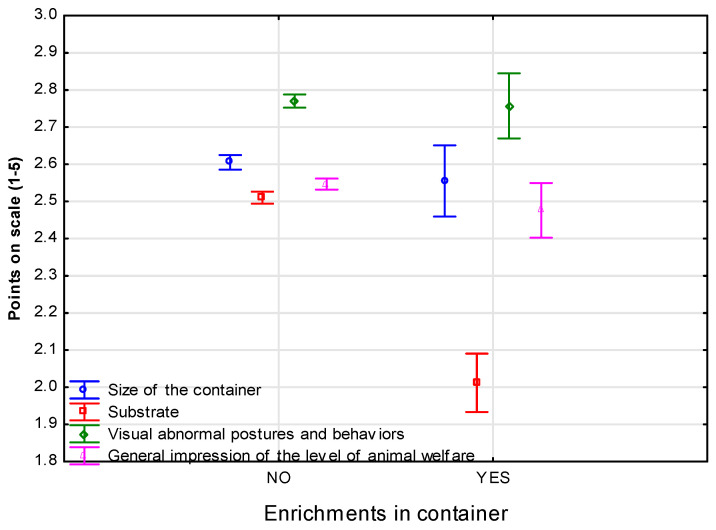
Mean scores on evaluation in case of presence of environmental enrichment in display containers. Five-point Likert scale with one representing the lowest rating and five denoting the highest; for descriptive statistics, mean arithmetic (M) with standard deviation (SD) was used.

Figure 4 shows how containers were rated depending on whether they were equipped with environmental enrichment or not. Interestingly, the presence of environmental enrichments in the containers did not cause them to receive higher scores during evaluation (Figure 4). The differences between the mean scores of the containers in terms of the presence of enrichments in them were statistically significantly different (F(4, 17,173) = 37.87, *p* < 0.001).

**Figure 5:**
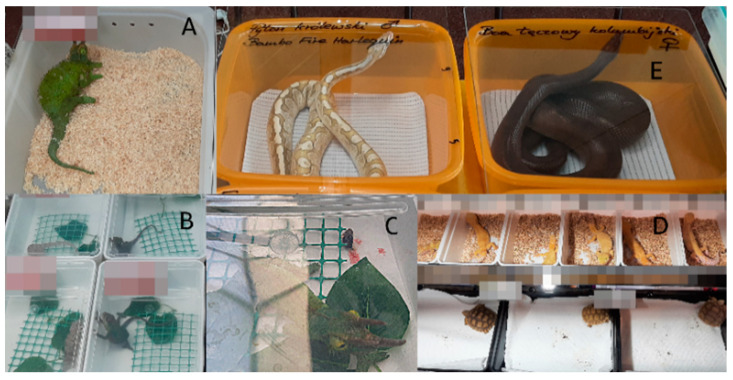
Examples of incorrect ways of presenting reptiles for sale at the examined fair (the breeders’ name was blurred on the photo). (**A**–**C**)—chameleons presented in horizontal containers: (**A**) *Triceros jacksonii* without environmental enrichment and with unsuitable substrate; (**B**) *Furcifer pardalis* had enrichment (for climbing) but flat container prevented them from climbing; paper towel is used as substrate; (**C**) *Triceros jacksonii* had enrichment; paper towel used as substrate; visibly bloody droppings, suggesting health problems. (**D**) Upper containers: *Eublepharis macularius* had insufficient substrate and cramped container; bottom container: *Centrochelys sulcata*, proper container size but inadequate substrate; (**E**) *Python regius* and *Epicrates cenchria maurus* kept in small containers, with inappropriate substrate and covered with an acrylic lid without any ventilation holes; snakes directed their nostrils to the micro-gap at the junction of the lid with the container, and it was suspected that there were was little air in the container.

Some of the tortoises were provided with substrate in the form of wooden cuttings or paper towels, which is inappropriate for them. In the case of *Centrochelys sulcata* turtles shown in Figure 5D, the vendor included an information leaflet stating their target size and body weight (up to 50 kg). Such information, despite being required at such events, is rare. It is important to pay attention to the small access to air in the containers housing animals. Usually, small circular vents are made in the containers; however, it was also observed that there were no vents at all in the containers. It has been observed, especially in the case of snakes and lizards, that they adopt a body position with their nostrils facing a source of fresh air (Figure 5E).

### 3.2. Testers Reliability

A comparison between individual testers as well as between replicates (trials) showed statistically significant differences (F(24, 59,893) = 575.62, *p* < 0.001; F(8, 34,344) = 36.723, *p* < 0.001, respectively). Pearson’s correlation coefficient showed that the assessments in each trial are mostly >0.8, indicating good reliability of the evaluators (Table 2).

A two-way ANOVA showed a significant difference between testers considering successive repetitions (tester*trial: F(48, 66,081) = 70.70, *p* < 0.001) (Table 3). Correlation coefficients examining the repeatability of individual testers are shown in Table 3. In assessing container size, tester No. 6 had the highest intra-rater reliability (mean r = 0.95), and tester No. 4 had the lowest (mean r = 0.75). Significant differences in the ratings were noted for visual abnormal postures and behavior, and the average correlation coefficient obtained ranged from 0.64 to 0.98.

**Figure 6:**
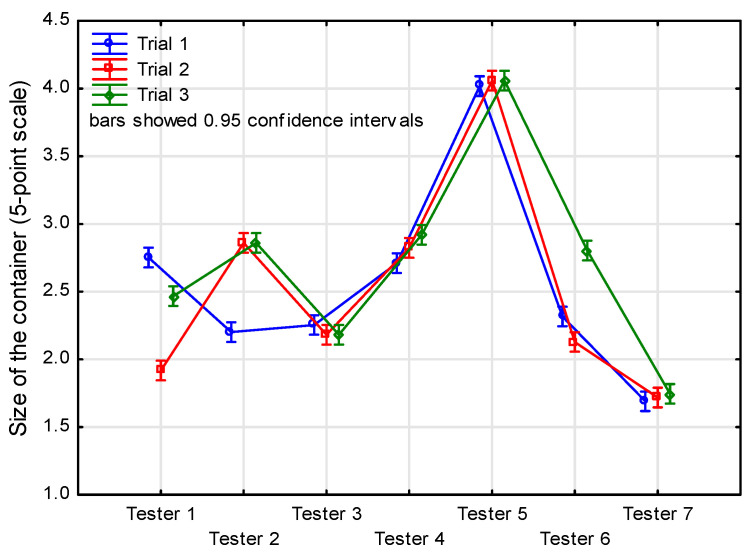
Results of container size evaluation according to tester and trial number.

**Figure 7:**
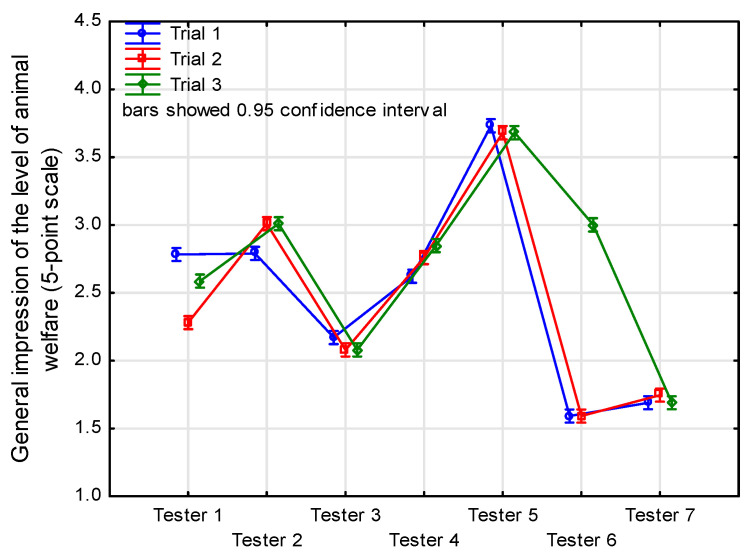
Result of general impression of the level of animal welfare according to tester and trial number.

Noticeably, depending on the feature being evaluated, testers had different perceptions. They were concerned about the container size (Figure 6), and tester 5 was rated higher than the others. In the case of the substrate used, the results of the ratings were more divergent but were in a similar range of 2.15–2.95 points; the ratings given in this measure were correlated among all testers at 0.78–0.87. The overall feelings about the welfare of the animals in the boxes ranged from 1.5 to 3.75 points, depending on the tester (Figure 7). This means that the testers considered the level of animal welfare at the time these photographs were taken to be average, or difficult to define clearly. Testers 6 and 7 were the most critical in this regard (No. 6), and they had a relatively poor repeatability of evaluation (0.48–0.65).

## 4. Discussion

The results showed that the ways animals are exposed during fairs can negatively affect their welfare. The display containers tended to be insufficiently sized for the animals, especially for snakes, chameleons, monitor lizards, and salamanders. Visual signs of abnormal behavior were also observed, with animals attempting to gasp for air (inadequate ventilation of containers, see Figure 5E) [10]. It is also interesting to note that environmental enrichments in the containers did not raise the scores obtained, perhaps influenced by the small number of containers with environmental enrichment other than the substrate. Intra-rater reliability results have shown that a properly trained team provides the opportunity to obtain reliable, reproducible results when the features discussed earlier are assessed. Thus, the use of photography as a data source may find broader use in studies where it is impossible to devote a significant amount of time to proper measurements due to time constraints or a large number of animals to be seen in a short period [50,52].

One of the main problems that animal fairs face relates to how animals are presented and the size of exhibition containers. Recently, in Poland, Stowarzyszenie Terrarystystów Polskich (STP, ang. the Association of Polish Terrarium Keepers; https://stp.net.pl/ accessed on 16 January 2024) signed an agreement with six organizers of exotic animal fairs held in Poland in which the organizers agreed to comply with the codes of good practice on how to prepare the animals for the fair (unfortunately, not all organizers of such events in Poland have cooperated, but they are a minority). It is worth noting that the results were obtained before the preparation of this document. The rules of Terraristika Hamm and Terraria Houten [41,42] now include minimum container sizes: 150% of the body length for lizards and amphibians, 33% of the total length (TL) for snakes, and 200% of the carapace length for tortoises and turtles. At least 50% of an animal housing has to be accessible for movement. Lizards and amphibians must be able to stretch. The STP has set requirements in this regard; for lizards and chameleons, the minimum length of display containers should be equal to the TL, including the tail; for long-tailed species (where the tail accounts for 70% of the total length), it should be a minimum of 50% of the TL. On the other hand, snakes should be provided with a tank that is 1/3 of the TL of the snake. The use of <1 × TL (total length of the snake) enclosures comes mainly from two non-scientific sources based on the suggestions of snake breeders and includes justification relating to personal opinion and traditional practice [57]. Moreover, this approach is still common among keepers of many reptile species [6], and a survey [58] found that 54.7% of snakes were kept in enclosures shorter in length or height than their body length. No scientific studies have shown that smaller and unnatural enclosures are not harmful to animals [57]. Instead, studies have shown that keeping snakes in large tanks (enclosure sizes that allow snakes to stretch fully) benefits their health and welfare [19]. This discussion continues between the breeding community, pro-animal organizations, and scientists [59]. For turtles and tortoises, the same as at Hamm and Houten, the longest side of the container should be 200% of the carapace length, and the shorter one should be equal to the carapace length. It is recommended that lizards and snakes be kept singly. Turtles and tortoises can be kept singly or in groups. The STP also notes other requirements related to the use of environmental enrichment, e.g., for arboreal species, the use of vertical tanks and the use of branches for climbing. At the same time, it is not recommended to expose turtles in water-filled containers, and in the case of amphibians, the container should be lined with a substrate that provides optimal humidity. However, exposing *Abystoma mexicanum* amphibians (commonly known as the axolotl) at such events is forbidden due to the low possibility of providing them with an adequate water temperature (less than 18 °C).

The size of the container and equipment used appear critical in avoiding this exposure to unfavorable conditions. Such exotic animal fairs are short, usually one-day events; once the vendor gets to the site, it does not take more than 24 h. Therefore, they can be treated as short-term events [15]. Animals offered for sale at such short-term events are often subjected to multifactorial stressors of varying degrees of severity (e.g., intensive rearing in a rack system, capture, placement in display containers, transportation, display, transport to a new keeper, and entry into a new tank with a different degree of environmental enrichment). In this situation, the body’s response to emerging stressors may be complex; depending on the intensity of the stimulus, the discharge of stress hormones may vary [60]. Some amphibians and reptiles have the ability to modulate their adrenal cortex’s response to stress; however, other species do not [61], which aligns with the visual expression of reduced welfare resulting from stress [60,62]. These rapid, physiological changes in color enable animals to respond to periodic sensory deprivation or temporal variation in background color, changes in thermal requirements during the day, and the visual presence of a threat (humans) [62,63]. Coloration changes during fairs are usually observed in lizards (e.g., *Phelsuma*, *Anolis* and chameleons). Short-term conditions should be interpreted as an unavoidable minimum period during which, for overriding practical reasons, the environment may not fully comply with modern welfare rules [11,14]. The EU is working to introduce new regulations to improve animal welfare. Among other things, they pay attention to the transport of animals: travel times will be shortened, minimum space for the different animals will be increased and adapted to each species, and they emphasize the importance of responsible ownership of any pet animal [64].

Another important point worth discussing is sharing relevant information about the animal between the seller and the new owner. Containers should be labeled on the species, origin (trapping and captive-born), sex (if identifiable), degree of difficulty in maintaining and handling the animal, and recommended species for beginners [65,66,67]. Organizers should use their events not only for trade but also for education [68,69]. Some of the vendors provide brief information in flyers and on container labels, where the buyer will find basic information about the species and help them meet specific husbandry requirements for species. For animal well-being, it should be assumed that potential new owners have generalized deficiencies in knowledge of the biology and breeding of exotic animals; thus, the transfer of basic knowledge should take place at the time of handing over the animal to a new owner [70,71,72]. Purchasing exotic pets without prior substantive knowledge can be motivated by status factors, narcissistic and borderline personality traits, ostentation, social recognition, conformism and materialistic indulgence, or done on impulse [9,67,73,74]. Even if the new owner shows an interest in the species purchased, this is not synonymous with their sensitivity to the needs and general level of welfare [22,75]. Knowledge in the field of veterinary medicine, keeping exotic animals, for example, does not transfer to expertise in keeping reptiles and leads to various complications [33,72,76,77].

At Polish fairs, there are organizations (associations, foundations, shelters, e.g., Epicrates Foundation, Help4Herps Foundation, and STP) aiming to educate the public about environmental requirements, nutrition, and proper care and demonstrate the consequences of inadequate conditions for reptiles or amphibians (photo shows of animals with metabolic bone disease, rickets, etc.). When creating the regulations for such events, it is worth writing about the necessary presence of the educational aspect, which can contribute to providing appropriate conditions for purchased animals in the future.

Despite the organizers’ assurances that no animals may be sold in containers that do not meet the requirements of the regulations, at the fair we evaluated, the conditions provided for the animals were at an average level.

### Limitations of the Study

Some limitations of this work are worth emphasizing. The evaluation of all measures associated with having boxed animals on sale (i.e., the size of the boxes, the substrate used, presence of abnormal behavior, and level of welfare) was determined based on the objective assessment of each tester, as described in the Materials and Methods. Moreover, the measures were evaluated based on photographs taken 1 h before the opening of the fair, where animals were photographed in a specific position in the box (e.g., at a vent hole, digging in the substrate, snakes stacked along the walls of the box with a 90-degree bend in their spines, etc.) and at a specific time. Perhaps the same animals photographed at different times could be evaluated differently, especially regarding the presence of abnormal behavior and the overall impression of the level of welfare. Another consideration is the number of animals. Many vendors put only a portion of the available animals on display at the fair, with the remainder kept in styrofoam boxes and added to them as sales progress. Therefore, the number of animals at the fair may have been higher than we determined. Using only a 5-degree scale may not have given the experts enough opportunity to fully determine their feelings about the animal they were observing and to properly assess the parameters under study. This study was done before STP introduced its pre-event audits for proper animal display. It will be followed up with another study that will compare whether the introduction of restrictions and actual auditing of their application will result in better presentation of animals at such events resulting in improved animal welfare.

## 5. Conclusions

Problems involving maintaining the appropriate conditions and proper welfare of exotic animals at fairs have been noted and addressed by numerous authors. The entire reptile and amphibian husbandry community should focus on improving the requirements at the fairs. Improving the well-being of animals, in this case, can be achieved with strict regulations, but only to minimal standards. More research is needed on whether such events are actually stressful for animals and whether it affects their health. Stress is inevitable and not mutually exclusive to good welfare. It would be advisable to consider the creation of systemic solutions such as those proposed by the RSCA or STP catalog of good practices, in which sellers will find the specific conditions to be provided for exotic animals when traded. It is crucial to involve not only animal ethologists and animal welfare specialists in this discussion but also herpetological organizations, the breeding community, and vendors to establish guidelines for displaying animals at fairs, taking into account their expertise and perspectives. In addition, widespread, free, and mandatory education campaigns for exhibitors on these requirements and their importance in maintaining animal welfare should be introduced. Banning trade events, is not the solution. Perhaps making exhibitors’ ability to exhibit at trade shows conditional on receiving such training would significantly reduce the shortcomings associated with inadequate conditions for animals at trade shows, sensitize them to the needs of animals during this time, and improve the overall level of welfare.

## Figures and Tables

**Table 1:** Scoring with consideration of the systematic division of animals. Five-point Likert scale with one representing the lowest rating and five denoting the highest; for descriptive statistics, mean arithmetic (M) with standard deviation (SD) was used.

	Statistics	Snakes N = 279	Lizards * N = 308	Chameleons * N = 56	Turtles and Tortoises N = 62	Amphibians N = 113
Size of the container	F(4, 17,173) = 549.52, *p* < 0.001	2.07 ^A^ ± 0.02	2.74 ^A^ ± 0.01	2.46 ^A^ ± 0.05	3.37 ^ACD^ ± 0.03	3.09 ^AC^ ± 0.02
Substrate	F(4, 17,173) = 165.23, *p* < 0.001	2.52 ^A^ ± 0.13	2.57 ^A^ ± 0.01	2.04 ^BC^ ± 0.04	2.90 ^B^ ± 0.03	2.16 ^BC^ ± 0.02
Visual abnormal postures and behaviors	F(4, 17,173) = 306.22, *p* < 0.001	2.38 ^A^ ± 0.01	2.99 ^B^ ± 0.01	2.72 ^BC^ ± 0.04	2.68 ^BC^ ± 0.03	3.11 ^BCD^ ± 0.02
The general impression of the level of animal welfare	F(4, 17,173) = 190.99, *p* < 0.001	2.30 ^A^ ± 0.01	2.70 ^B^ ± 0.01	2.19 ^AB^ ± 0.04	2.74 ^B^ ± 0.03	2.67 ^B^ ± 0.02

Mean row values with different letter designations are statistically different at *p* < 0.01 (Tukey test for different N); * chameleons were separated from the lizard group due to significant differences in their needs and welfare levels.

**Table 2:** The test–retest (of 3 trials) reliability by using the Pearson correlation coefficient.

	Pearson’s Correlation Coefficient			
Trial	Size of the Container	Substrate	Visually Abnormal Postures and Behaviors	General Impression
1 vs. 2	0.84	0.85	0.81	0.82
2 vs. 3	0.87	0.87	0.89	0.69
1 vs. 3	0.87	0.78	0.89	0.66

**Table 3:** Tester reliability.

		Pearson Correlation Coefficient			
Tester	Trial Compared	Size of the Container	Substrate	Visual Abnormal Postures and Behaviors	General Impression
1	1 vs. 2	0.78	0.79	0.65	0.53
	2 vs. 3	0.90	0.80	0.65	0.46
	1 vs. 3	0.76	0.90	0.94	0.84
2	1 vs. 2	0.78	0.69	0.62	0.68
	2 vs. 3	0.83	0.72	0.71	0.72
	1 vs. 3	0.79	0.68	0.65	0.67
3	1 vs. 2	0.87	0.86	0.82	0.74
	2 vs. 3	0.87	0.90	0.84	0.72
	1 vs. 3	0.89	0.84	0.79	0.75
4	1 vs. 2	0.68	0.40	0.56	0.56
	2 vs. 3	0.76	0.68	0.61	0.67
	1 vs. 3	0.81	0.52	0.74	0.66
5	1 vs. 2	0.98	0.97	0.98	0.72
	2 vs. 3	0.89	0.90	0.99	0.73
	1 vs. 3	0.98	0.92	0.98	0.68
6	1 vs. 2	0.91	0.52	0.97	0.65
	2 vs. 3	0.84	0.32	0.94	0.48
	1 vs. 3	0.74	0.26	0.95	0.56
7	1 vs. 2	0.94	0.98	0.93	0.87
	2 vs. 3	0.88	0.96	0.89	0.95
	1 vs. 3	0.95	0.98	0.93	0.86

## Data Availability

The data presented in this study are available upon request from the corresponding author.

## Supplementary Materials

The following supporting information can be downloaded at: https://www.mdpi.com/article/10.3390/ani14131872/s1, Supplement S1: Supplement to the material and methods chapter in the preparation of testers for the evaluation of fair photos, and environmental and welfare requirements. Refs. [5,6,10,17,18,19,23,24,39,54,58,59,60,67,78,79,80,81,82,83,84,85,86,87,88] are cited in Supplementary Materials.
