# Supplementary material for: Assessment of Animal Welfare at an Exotic Animal Fair in Poland: A Focus on the Quality of Exhibition Containers for Reptiles and Amphibians
Source: Animals (Basel). 2024 Jun 25;14(13):1872. doi: 10.3390/ani14131872 (PMC11240396; doi:10.3390/ani14131872)
Supplement: Supplementary file 1 [file animals-14-01872-s001.zip › animals-2894471-supplementary.pdf]

Supplementary Material S1: Supplement to the material and methods chapter in the preparation of testers for the evaluation of fair photos, and environmental and welfare requirements.

Pre-study regime for evaluators

List of scientific papers that the assessor read and understood before making evaluation of the chosen measures (Size of the container, Substrate, Visual abnormal postures and behaviors, The general impression of the level of animal welfare) in the studied exotic animal fair in Poland.

1. Alligood, C., & Leighty, K. (2015). Putting the "E" in SPIDER: Evolving trends in the evaluation of environmental enrichment efficacy in zoological settings. *Anim. Behav. Cogn*, *2*(3), 200-217.
2. Arena, P. C., Crawford, M., Forbes, N. A., Frye, F. L., Grant, R., Howell, T., ... & Whitehead, M. (2018). The need for snakes to fully stretch. *The Veterinary Record*, *183*(21), 661.
3. Auliya, M.; Altherr, S.; Ariano-Sánchez, D.; Baard, E.; Brown, C.; Brown, R.; Cantu, C.; Gentile, G.; Gildenhuys, P.; Henningheim, E.; et al. Trade in live reptiles, its impact on wild populations, and the role of the European market. Biol. Conserv. 2016, 204, 103–119.
4. Bacon, H. (2018). Behaviour-based husbandry—A holistic approach to the management of abnormal repetitive behaviors. *Animals*, *8*(7), 103.
5. Bashaw, M. J., Gibson, M. D., Schowe, D. M., & Kucher, A. S. (2016). Does enrichment improve reptile welfare? Leopard geckos (Eublepharis macularius) respond to five types of environmental enrichment. *Applied animal behaviour science*, *184*, 150-160.
6. Benn, A.L.; McLelland, D.J.; Whittaker, A.L. A Review of Welfare Assessment Methods in Reptiles, and Preliminary Application of theWelfare Quality® Protocol to the Pygmy Blue-Tongue Skink, Tiliqua adelaidensis, Using Animal-Based Measures. Animals 2019, 9, 27.
7. Burghardt, G.M. Keeping reptiles and amphibians as pets: Challenges and rewards. Vet. Rec. 2017, 181, 447–449.
8. Cargill, B. M., Benato, L., & Rooney, N. J. (2022). A survey exploring the impact of housing and husbandry on pet snake welfare. *Animal Welfare*, *31*(2), 193-208.
9. D'Cruze, N., Paterson, S., Green, J., Megson, D., Warwick, C., Coulthard, E., ... & Carder, G. (2020). Dropping the ball? The welfare of ball pythons traded in the EU and North America. *Animals*, *10*(3), 413.
10. Green, J., Coulthard, E., Megson, D., Norrey, J., Norrey, L., Rowntree, J. K., ... & D'Cruze, N. (2020). Blind trading: a literature review of research addressing the welfare of ball pythons in the exotic pet trade. *Animals*, *10*(2), 193.
11. Hoehfurtner, T., Wilkinson, A., Walker, M., & Burman, O. H. (2021). Does enclosure size influence the behaviour & welfare of captive snakes (Pantherophis guttatus)?. *Applied Animal Behaviour Science*, *243*, 105435.
12. Howell, T. J., Warwick, C., & Bennett, P. (2022). Pet management practices of frog and turtle owners in Victoria, Australia. *Veterinary Record*, *191*(12),
13. Martínez Silvestre, A. How to assess stress in reptiles. J. Exotic Pet Med. 2014, 23, 240–243.
14. Michaels, C. J., Downie, J. R., & Campbell-Palmer, R. (2014). The importance of enrichment for advancing amphibian welfare and conservation goals. *Amphibian Reptile Conservation*, *8*, 7-23.

15. Rose, P. E., Nash, S. M., & Riley, L. M. (2017). To pace or not to pace? A review of what abnormal repetitive behavior tells us about zoo animal management. *Journal of Veterinary Behavior*, *20*, 11-21.
16. RSPCA guidelines for different popular pet reptiles Available online: https://www.rspca.org.uk/adviceandwelfare/pets/other
17. Schuppli, C.A.; Fraser, D.; Bacon, H.J. Welfare of non-traditional pets. Rev. Sci. Tech. 2014, 33, 221–231.
18. Warwick, C., Jessop, M., Arena, P., Pilny, A., & Steedman, C. (2018). Guidelines for inspection of companion and commercial animal establishments. *Frontiers in veterinary science*, *5*, 151.
19. Warwick, C., Pilny, A., Steedman, C., Howell, T., Martínez-Silvestre, A., Cadenas, V., & Grant, R. (2023). Mobile zoos and other itinerant animal handling events: current status and recommendations for future policies. *Animals*, *13*(2), 214.
20. Warwick, C.; Arena, P.; Lindley, S.; Jessop, M.; Steedman, C. Assessing reptile welfare using behavioural criteria. InPractice 2013, 35, 123–131.
21. Warwick, C.; Arena, P.; Steedman, C. Spatial considerations for captive snakes. J. Vet. Behav. Clin. Appl. Res. 2019, 30, 37–48.
22. Warwick, C.; Jessop, M.; Arena, P.; Pliny, A.; Nicholas, E.; Lambiris, A. Future of keeping pet reptiles and amphibians: animal welfare and public health perspective. Veterinary Record. 2017, 181, 454–455.
23. Warwick, C.; Steedman, C.; Jessop, M.; Arena, P.; Pilny, A.; Nicholas, E. Exotic pet suitability: Understanding some problems and using a labeling system to aid animal welfare, environment, and consumer protection. J. Vet. Behav. 2018, 26, 17–26.
24. Whitehead, M.L. Factors contributing to poor welfare of pet reptiles. Testudo 2018, 8, 47–61
25. Zieliński, D. (2023). The Effect of Enrichment on Leopard Geckos (Eublepharis macularius) Housed in Two Different Maintenance Systems (Rack System vs. Terrarium). *Animals*, *13*(6), 1111.

The environmental criteria for evaluators:

- Size of the container –the ratio of the size of the animal to the length of the longest side of the container was visually assessed; container orientation, horizontal for terrestrial species, vertical for arboreal, climbing species
- Presence, type and adaptation of substrate type to the needs of the species;
- Whether the animal was kept at the right humidity for its species (higher humidity for amphibians and tropical species, low humidity for dryland, steppe, desert species)
- Number of animals per container;

The welfare assessment criteria:

- If any signs of low welfare issues were observed ratings of 1-2 were given, e.g. being at the air vent, unnatural body position, adjusting the body to the contour of the container (snakes), color change to dark (geckos, chameleons), observed health issues: bite marks, moulting residue, injuries, tail end missing, bloody droppings, no additional environmental enrichment, places to hide and the possibility of climbing for arboreal species; the presence of substrate or paper towel was not considered as enrichment;
- A middle score (3) was given when the evaluator was not sure if the animal's body position was abnormal and could indicate some welfare issues
- If the animal was photographed in a normal body position, not indicative of any negative behavior and no visual health issues were observed then ratings of 4 and 5 were given.
